# Tracking Drug Resistance in *Plasmodium falciparum*: Genetic Diversity of Key Resistance Markers in Brazilian Malaria Hotspots

**DOI:** 10.3390/ijms26135977

**Published:** 2025-06-21

**Authors:** Rebecca de Abreu-Fernandes, Lucas Tavares de Queiroz, Natália Ketrin Almeida-de-Oliveira, Aline Rosa de Lavigne Mello, Jacqueline de Aguiar Barros, Lilian Rose Pratt-Riccio, Gisely Cardoso de Melo, Patrícia Brasil, Cláudio Tadeu Daniel-Ribeiro, Didier Menard, Maria de Fátima Ferreira-da-Cruz

**Affiliations:** 1 Laboratório de Pesquisa em Malária, Instituto Oswaldo Cruz, Fundação Oswaldo Cruz (Fiocruz), Rio de Janeiro 21041-361, Brazil; rebeccasantos@aluno.fiocruz.br (R.d.A.-F.); lucasqueiroz@aluno.fiocruz.br (L.T.d.Q.); nataliaketrin@gmail.com (N.K.A.-d.-O.); alinelavigne@uol.com.br (A.R.d.L.M.); barros.jacqueline@gmail.com (J.d.A.B.); riccio@ioc.fiocruz.br (L.R.P.-R.); malaria@fiocruz.br (C.T.D.-R.); 2 Centro de Pesquisa, Diagnóstico e Treinamento em Malária (CPD-Mal), Reference Laboratory for Malaria in the Extra-Amazonian Region for the Brazilian Ministry of Health, Secretaria de Vigilância Sanitária & Fiocruz, Rio de Janeiro 21041-361, Brazil; patricia.brasil@ini.fiocruz.br; 3 Núcleo de Controle da Malária, Departamento de Vigilância Epidemiológica, Coordenação Geral de Vigilância em Saúde, SESAU-RR, CIEVS/SVS/SMSA-BV, Boa Vista 69305-080, Brazil; 4 Fundação de Medicina Tropical Dr. Heitor Vieira Dourado (FMT-HVD), Manaus 69040-000, Brazil; cardosogisely@gmail.com; 5 Universidade do Estado do Amazonas (UEAM), Manaus 69010-455, Brazil; 6 Laboratório de Pesquisa Clínica em Doenças Febris Agudas, Instituto Nacional de Infectologia Evandro Chagas, Fiocruz, Rio de Janeiro 21040-360, Brazil; 7 Malaria Parasite Biology and Vaccines Unit, Institut Pasteur, Université Paris Cité, F-75015 Paris, France; dmenard@pasteur.fr; 8 Malaria Genetics and Resistance Team (MEGATEAM), UR 3073—Pathogens Host Arthropods Vectors Interactions, Université de Strasbourg, F-67000 Strasbourg, France; 9 CHU Strasbourg, Laboratory of Parasitology and Medical Mycology, F-67081 Strasbourg, France; 10 Institut Universitaire de France (IUF), F-75231 Paris, France

**Keywords:** *Plasmodium falciparum*, antimalarial drugs, resistance, Brazilian endemic areas

## Abstract

Malaria remains a health problem, with *Plasmodium falciparum* accounting for 96% of cases in Africa and 15% in Brazil. The growing threat of drug resistance to artemisinin-based combination therapies (ACTs) jeopardizes progress toward elimination. This study examined *P. falciparum* samples collected from 141 patients in Brazil (2013–2023) by PCR and DNA sequencing to identify single-nucleotide polymorphisms in the *pfcrt*, *pfmdr1*, and *pfk13* genes. Half of the samples carried the **S**VMN**T**MCGI haplotype in *pfcrt*, and none of the samples showed C350**R** mutations. In *pfmdr1*, the NY**CDY** haplotype was dominant (70%), with low occurrences of N86**Y** (4%) and no Y184**F** polymorphisms. No mutations linked to artemisinin partial resistance were detected in *pfk13*. Only one Amazonas sample exhibited wild-type haplotypes across all genes. Genetic diversity was more pronounced in *pfcrt* than *pfmdr1*, reflecting selective drug pressure. Significant linkage disequilibrium (LD) was observed within *pfcrt* (C72S and K76T) and *pfmdr1* (S1034C and N1042D), but not between the two genes. The absence of *pfk13*-resistant mutations and the low prevalence of key *pfmdr1* markers support the efficacy of ACTs. The persistence of diverse haplotypes and intragenic LD reflects ongoing drug pressure, underscoring the need for continuous genetic surveillance to anticipate emerging resistance.

## 1. Introduction

Malaria remains a major parasitic disease with high mortality rates, particularly in tropical and subtropical regions. Despite being a life-threatening infectious disease, malaria still affects millions globally [1]. In 2023, approximately 263 million malaria cases were reported, resulting in 619,000 deaths [2]. The lethal parasite *Plasmodium falciparum* accounted for 15% of the Brazilian cases [3]. Over the past two decades, substantial progress has been made in reducing malaria incidence, primarily through the widespread adoption of artemisinin-based combination therapies (ACTs) as the first-line treatment for uncomplicated *Plasmodium falciparum* malaria in endemic regions [1]. In Brazil, current treatment guidelines recommend either artemether/lumefantrine (AM–LM) or artesunate/mefloquine (AS–MQ), combined with a single dose of primaquine (SLDP), depending on regional availability [4]. However, this progress is under threat due to the emergence and spread of antimalarial drug resistance.

The *P. falciparum* drug resistance poses a significant challenge for malaria control programs. Chloroquine resistance (CQR) in the late 1950s marked a pivotal moment, seriously undermining global disease control efforts [5]. Since then, resistance has developed to multiple antimalarial drugs, including sulfadoxine–pyrimethamine (SP) [6], mefloquine (MQ) [7], and, more recently, artemisinin (ART) [8]. Documented resistance hotspots in Southeast Asia and South America further emphasize the urgent need for continuous surveillance [9,10]. Recently, the *P. falciparum* kelch propeller domain on chromosome 13 (*pfk13*) has been extensively studied and identified as a key marker for ART partial resistance (ART-R) [11,12,13,14]. To date, more than 200 SNPs have been identified in *pfk13*, though only 13 (F446**I**, N458**Y**, M476**I**, Y493**H**, R539**T**, I543**T**, P553**L**, R561**H**, P574**L**, C580**Y**, R622**I**, C469**Y**, and A675**V**) are officially linked to ART-R [14]. While ART-R is an emerging global threat, CQR remains persistent and particularly problematic in South America due to its distinct molecular mechanisms.

CQR is strongly associated with the K76**T** polymorphism and the **S**VMN**T** haplotype of the *pfcrt* gene, which have been detected globally but are particularly prevalent in South America [15]. Although the cessation of CQ use has led to the re-emergence of the wild-type allele and restored drug susceptibility in many regions, the persistence of the K76**T** allele in Brazil suggests ongoing selective pressure or other factors maintaining this polymorphism [16]. In South America, the reversal of CQR has been attributed to the *pfcrt* C350**R** mutation [9,17]. However, this mutation has not yet been investigated in Brazilian isolates, emphasizing the need to examine whether it is circulating to support an eventual reintroduction of CQ as a treatment option.

The *pfmdr1* (multidrug resistance 1) gene modulates *P. falciparum* susceptibility to key heme-binding antimalarials, including CQ, MQ, LM, and ART [18]. Specific point mutations drive its influence on drug resistance and have been linked to treatment failures in ACTs [19,20]. Five significant SNPs have been identified in *pfmdr1*: N86**Y**, Y184**F**, S1034**C**, N1042**D**, and D1246**Y**, each with distinct effects on drug efficacy and resistance patterns. The N86**Y** mutation is linked to CQR and the ACT partner drug amodiaquine (AQ), and enhances the parasite’s sensitivity to MQ, LM, and dihydroartemisinin (DHA) [18,19,20]. In contrast, the Y184**F** mutation has been associated with reduced LM sensitivity [19]. Although S1034**C**, N1042**D**, and D1246**Y** do not independently confer phenotypic resistance, they contribute to the emergence of resistant haplotypes when combined with other mutations. By modifying drug transport kinetics, these variants can alter the parasite’s susceptibility to treatment and potentially compromise the efficacy of ACT regimens [18]. Their phenotypic impact is primarily determined by the specific genetic contexts in which they arise within the parasite genome. Notably, haplotypes such as **YYY** (86**Y**/184**Y**/1246**Y**) have been shown to modulate resistance and sensitivity patterns to antimalarial therapies, underscoring the complex genetic interactions that shape treatment outcomes [21,22].

Mutations in the *pfcrt* gene and those in *pfmdr1* further enhance resistance to antimalarial drugs by creating synergistic effects that impair drug efficacy [21]. The wild-type K76 *pfcrt* allele and the N**F**D *pfmdr1* haplotype (N86/184**F**/D1246) have been associated with decreased sensitivity to LM [20]. These alleles are frequently selected under AM–LM drug pressure [19,20,21], a first-line ACT therapy widely used in Brazil [4]. Moreover, the K76**T** mutation in *pfcrt*, when combined with the **YYY** haplotype in *pfmdr1*, has been strongly linked to clinical treatment failure (i.e., recrudescence following artesunate–amodiaquine, AS–AQ therapy) [21,22]. Monitoring these genetic markers is essential to predict therapeutic failure and preserve the long-term efficacy of ACTs. Identifying key haplotypes and their association with specific resistance phenotypes enables the development of more targeted treatment regimens and informs public health interventions to mitigate the spread of resistance.

Furthermore, understanding the genetic diversity of resistance-associated genes is crucial for identifying emerging mutations that could undermine the effectiveness of ACT. Linkage disequilibrium (LD) analysis enhances this understanding by revealing non-random associations between mutations, clarifying how resistance variants co-evolve and spread within parasite populations. By mapping the genetic structure and transmission dynamics of resistance markers, LD analysis can predict how multidrug resistance propagates, thereby guiding the development of targeted control strategies.

Thus, this study investigated the SNPs in key resistance-associated *P. falciparum* genes (*pfcrt*, *pfmdr1*, and *pfk13*) as well as their genetic diversity to provide critical insights into resistance dynamics and inform evidence-based treatment strategies.

## 2. Results

### 2.1. Prevalence of Drug Resistance Markers

Analysis of the sequences from 141 samples revealed no known mutations associated with ART-R in the *pfk13* gene. Haplotype analysis indicated that the wild-type profile predominated among all tested samples. Among the 141 *P. falciparum* samples, mutations in the codons C72**S** and K76**T** were identified in 99% (139 out of 141) of the samples, while mutations in codon I356**L** were detected in 40% (56 out of 141). No mutations were observed in codons M343**L** or G353**V**. Notably, the *pfcrt* C350**R** mutation, which has recently been linked to a reverse phenotypic response involving CQ sensitivity and piperaquine resistance, was not detected. Haplotype analysis also showed that the most common variant was the double mutant haplotype (**S**VMN**T**MCGI), present in 59% (83 out of 141) of isolates. The triple mutant haplotype (**S**VMN**T**MCG**L**) was also identified in 40%, while the wild-type haplotype (CVMNKMCGI) was found in only 1% of the isolates (Table 1).

For the *pfmdr1* gene, the analysis revealed highly prevalent mutations in codons S1034**C** (81%), N1042**D** (81%), and D1246**Y** (71%). The N86**Y** polymorphism was identified in only five isolates (4%), one from Roraima and four from Amazonas states. None of the isolates carried the Y184**F** mutation in this study. Five *pfmdr1* haplotypes at the N86**Y**–Y184**F**–S1034**C**–N1042**D**–D1246**Y** *loci* were identified. The NY**CDY** haplotype was observed in 70% (98/141) of the isolates, while the NYSND wild-type haplotype was documented at a frequency of 13% (19/141) (Table 2).

### 2.2. Combined Haplotypes of pfk13, pfcrt, and pfmdr1

The *pfk13*, *pfcrt*, and *pfmdr1* genes were genotyped successfully in all 141 cases, and the combined haplotypes are summarized in Table 3. They had 11 combined haplotypes, predominated by wild-type + **S**VMN**T**MCGI + NY**CDY** and wild-type + **S**VMN**T**MCG**L** + NY**CDY**, with 38% and 31% prevalence, respectively. Only one Amazonas sample carried a wild-type haplotype profile for the SNPs of the three analyzed genes (Table 3).

Complementary notification and clinical follow-up data were retrieved from the SIVEP-Malaria system for patients from Roraima and Amazonas. No cases of recrudescence were identified among the patients with available SIVEP records or those followed at CPD-Mal, as none presented a recurrence of *P. falciparum* infection within 28 days, or up to 42 days in the cases of suspected treatment failure [4].

### 2.3. Genetic Diversity and Linkage Disequilibrium (LD) Analysis

The analysis of genetic diversity revealed that *pfcrt* showed the highest genetic variation, exhibiting significantly greater nucleotide and haplotype diversity compared to *pfmdr1*, while *pfk13* remained monomorphic with no detected polymorphisms (Table 4). Neutrality tests indicated negative Tajima’s D values for exon 2 of *pfcrt* (*−*0.39181|*p*  >  0.10), suggesting potential population expansion or purifying selection. In contrast, positive values were observed for exon 10 of *pfcrt* (1.72185|*p*  >  0.10), indicating balancing selection. Similarly, *pfmdr1* exhibited negative values in its initial region (*−*0.66673|*p*  >  0.10) and positive values in its terminal region (1.58461|*p*  >  0.10), reflecting distinct selective pressures across the gene (Table 4).

**Figure 1:**
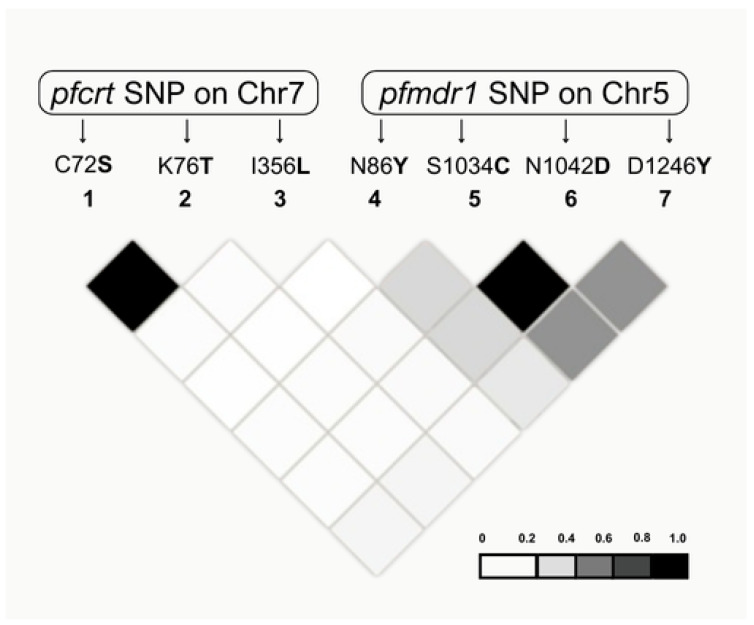
LD plot between SNPs of the *pfcrt* and *pfmdr1* genes in *P. falciparum* isolates from the Brazilian endemic areas. Each diamond represents the r^2^ value between two SNPs, and darker shades indicate stronger LD (r^2^ values closer to 1 reflect stronger linkage).

To evaluate linkage disequilibrium (LD) within and between resistance haplotypes, the LD pattern for each SNP in the *pfcrt* and *pfmdr1* genes was analyzed (Figure 1). Statistically significant intragenic associations were identified between the SNPs C72**S** and K76**T** in *pfcrt* and between 1034**C** and 1042**D** in *pfmdr1*. However, no significant intergenic LD association was detected between the two genes (Figure 1).

## 3. Discussion

ACT therapy is the World Health Organization’s recommended treatment for uncomplicated *P. falciparum* malaria in all malaria-endemic regions [2,23]. Artemisinin and its derivatives quickly reduce the majority of the parasite biomass. When combined with a long-acting partner drug, this strategy effectively eliminates any remaining parasites [4]. In 2006, the Brazilian government adopted this guideline as the first-line treatment for all diagnosed cases of uncomplicated *P. falciparum* malaria. Currently, two ACTs are endorsed: AM-LM and AS–MQ [4]. Due to widespread resistance, CQ is no longer used to treat *P. falciparum* infections. However, CQ remains the first-line therapy for *P. vivax* malaria in Brazil, alongside primaquine [24].

A comprehensive understanding of the genetic factors that influence drug resistance is essential for addressing the decline in antimalarial effectiveness. Examining key resistance genes such as *pfk13*, *pfcrt,* and *pfmdr1* is crucial for revealing the molecular mechanisms that lead to therapeutic failure [17]. Ongoing genetic monitoring of these markers is crucial for maintaining the long-term efficacy of ACT regimens and for making timely adjustments to treatment strategies in response to emerging resistance trends. Consequently, this study assessed the prevalence of mutations associated with drug resistance in *P. falciparum* parasites from endemic regions in Brazil.

As seen in Southeast Asia, the *pfk13* gene is crucial due to its association with ART-R, particularly from SNPs in the propeller domain [13,25]. Thus far, no *pfk13* mutations linked to ART-R have been detected in Brazilian isolates [26,27,28,29], and our analysis confirmed the absence of mutations both in resistance-associated codons and other regions of the propeller domain, supporting the continued efficacy of ACTs in this country. However, emerging evidence from neighboring borders raises concerns about the spread of resistant parasite lineages. The *pfk13* C580**Y** mutation in Guyana, associated with in vitro resistance to ART, has been confirmed [28]. Additionally, the *pfk13* A481**V** mutation was reported in Manaus State, Brazil, though no clinical data were available to evaluate its impact [30]. Although this mutation is not classically associated with those described in Southeast Asia, emerging evidence suggests that other *pfk13* mutations may be present in South America [28,29,30]. For instance, the A504**D** mutation has been identified at low frequency in Colombia [31], reinforcing the need for sustained genomic surveillance to determine its potential impact on drug efficacy. Detecting these mutations underscores the risk of resistance spreading across borders, mainly due to migration driven by illegal gold mining between Guyana, Brazil, and Venezuela [29,32]. This highlights the need for enhanced regional surveillance and coordinated monitoring to prevent the spread of ARTR *P. falciparum* strains in the Amazon basin.

Likewise, the *pfcrt* mutation K76**T**, commonly linked with other non-synonymous mutations at codons 72, 74, or 75, acts as the primary mediator of CQR [33]. Our analysis revealed a high prevalence of mutations in *pfcrt* codons 72–76, with the **S**VMN**T** haplotype present in 96% of isolates, aligning with previous studies in the Amazon Basin [16]. A greater prevalence of CQ-sensitive parasites was expected in Brazil, where CQ has not been utilized to treat uncomplicated falciparum malaria since the 1980s. However, the continued use of CQ for treating *P. vivax* malaria likely exerts selective pressure, contributing to the persistence of CQ-resistant *P. falciparum* populations [34,35]. Genetic diversity analysis further emphasized this ongoing selective pressure, revealing that *pfcrt* displayed the most variability, particularly in exon 2 (π = 0.00182; θ = 0.00249). In contrast, regions of Africa—where *P. falciparum* accounts for over 90% of malaria cases—have experienced significant declines in resistance due to reduced drug pressure, as CQ use has been restricted or considerably diminished [36].

We investigated mutations in exon 10 of the *pfcrt* gene (M343**L**, C350**R**, G353**V**, and I356**T**). All these mutations were absent in Brazilian samples, including *pfcrt* C350**R**, a key marker of CQ phenotypic reversion, and I356**T**, which is strongly associated with ART-R in Southeast Asia [30,37]. Instead, we detected a different substitution at this *locus*, I356**L**, in 40% of isolates. This mutation is commonly found in Latin America and seems more closely related to resistance against other antimalarials, such as CQ [17,38,39]. The absence of these key mutations, likely influenced by local drug usage patterns, contrasts with regions where piperaquine is commonly used and reflects the persistence of CQR in Brazilian isolates without phenotypic reversion.

The *pfmdr1* polymorphisms, including point mutations and gene amplification, affect *P. falciparum’s* sensitivity to several antimalarial drugs [18,40]. Our study found a high prevalence of the S1034**C**, N1042**D**, and D1246**Y** SNPs, with the NY**CDY** haplotype detected in 70% of the samples. This finding aligns with previous research from our team [26] and other studies in Brazil [35,41], suggesting that these resistance alleles may be approaching fixation. The N86**Y** mutation, linked to an increased susceptibility to MQ, LM, and DHA, as well as elevated resistance to CQ [18,19,20], was observed at a low frequency (4%). On the other hand, the Y184**F** mutation, associated with diminished LM sensitivity [19,40], was not present. These results aligned with earlier Brazilian reports, which showed low or absent frequencies of these mutations and may imply a reduced parasite susceptibility to AM–LM, MQ, and DHA [34,35].

The combined allelic distributions of *pfk13*, *pfcrt*, and *pfmdr1* among *P. falciparum* isolates revealed 11 distinct haplotype profiles. The wild-type *pfk13*, **S**VMN**T**MCG**L** *pfcrt,* and NY**CDY** *pfmdr1* were the most prevalent haplotypes. Even though all isolates contained the wild-type *pfk13*, its association with *pfcrt* (76**T**) and *pfmdr1* (86**N**, 184**F**, 1246**D***)* mutations may contribute to drug resistance [20,21]. These mutations are particularly notorious in African regions, where specific haplotypes, such as **S**VMN**T** + N**FCD**D, have been linked to recurrent parasitemia and treatment failure following AM–LM therapy [20,21]. However, in this study, neither the **S**VMN**T **+ N**FCD**D haplotype nor the **S**VMN**T** + **YYY** haplotype—typically associated with AS–AQ and CQ use [21,22]—was detected. These findings were further supported by the absence of recrudescence among patients with available follow-up at CPD-Mal and among those recorded in the SIVEP-Malaria system.

The absence of high-risk haplotypes indicated that current treatment regimens may limit their spread within the studied population. However, clinical efficacy, shown by the lack of recrudescence, does not eliminate the ongoing selection of resistant alleles. The presence of known resistance-associated mutations, primarily the *pfcrt* 76**T** and *pfmdr1* variants, suggested that the potential for resistance remains, especially if drug pressure continues. This finding was further supported by the genetic diversity analysis, which revealed distinct variation patterns among the three resistance-associated genes. The high nucleotide diversity (π = 0.00182, θ = 0.00249) and haplotype diversity (Hd = 0.146) observed in exon 2 of *pfcrt* indicated significant genetic differentiation, likely driven by prolonged CQ usage. In contrast, *pfmdr1* exhibited a varied diversity pattern, showing minimal variation in its initial region (π = 0.00012; Hd = 0.068) and increased diversity in its terminal region (π = 0.00112; Hd = 0.483), suggesting localized adaptation likely influenced by drug pressure. This variation was also noted in samples from Africa and India [42,43].

The lack of polymorphisms in *pfk13* was a significant finding, confirming the absence of ART-R-associated mutations in the analyzed Brazilian samples. This result aligned with the relatively low prevalence of ART-R observed outside South America [28,44]. The neutrality tests further clarified the selection dynamics influencing these resistance-associated *loci*. Negative Tajima’s D values for exon 2 of *pfcrt* (−0.39181) and the initial region of *pfmdr1* (−0.66673) indicated positive selection and the potential fixation of resistant variants, promoting the expansion of alleles related to drug resistance. In contrast, positive Tajima’s D values for exon 10 of *pfcrt* (1.72185) and the terminal region of *pfmdr1* (1.58461) suggested balancing selection, likely influenced by varying drug pressure, which preserves both resistant and sensitive alleles within the parasite population. This was further supported by positive Fu and Li’s D* and F* test values in the same regions, confirming evidence of ongoing selection pressure.

LD analysis is a molecular tool for identifying non-random associations among resistance markers, providing essential insights into the evolutionary pathways of drug-resistant haplotypes and their potential spread. Our LD analysis revealed significant intragenic associations between the C72**S** and K76**T** codons in *pfcrt* and between the 1034**C** and 1042**D** codons in *pfmdr1*, indicating coordinated selection within each gene. However, no significant intergenic associations were found, suggesting that the resistance mechanisms involving *pfcrt* and *pfmdr1* function independently and are affected by distinct selective pressures. These findings correspond with global studies emphasizing the critical role of intragenic associations in the persistence and evolution of drug-resistant populations [27,28,32].

The prevalence of the NY**CDY** haplotype in *pfmdr1* and the high frequency of *pfcrt* mutations indicated that selective pressure from ACT regimens, particularly AM–LM, continues to influence resistance dynamics and may contribute to the stabilization of these haplotypes within the parasite population. These findings underscore the need to integrate genetic surveillance into malaria control strategies, enabling the early detection of emerging resistance and guiding evidence-based treatment adjustments. Optimizing ACT allocation and strategically implementing alternative therapies in high-risk areas could be essential for maintaining treatment efficacy and mitigating the spread of resistance.

This study presented some limitations. Firstly, although all patients who attended at CPD-Mal were clinically and laboratory followed up, this study focused exclusively on the molecular markers of drug resistance without assessing the in vitro susceptibility of the parasites to antimalarial drugs. Second, while this study provided a comprehensive analysis of genetic markers, it did not include clinical data on treatment outcomes or parasite clearance rates. Third, although this study spanned a decade (2013–2023), no temporal trend analysis was conducted to assess the evolution of resistance markers over time. Fourth, the samples analyzed were collected from multiple endemic regions in Brazil; however, regional variations in the prevalence of resistance markers were not thoroughly explored.

In addition to monitoring established resistance markers, future studies should prioritize examining emerging genes, such as *pfcoronin* and the beta-propeller (BTB) domain of *pfk13.* Recent findings have linked mutations in *pfcoronin* to ART resistance in Southeast Asia, highlighting its potential role in the survival of parasites under drug pressure [45]. Similarly, the BTB domain of *pfk13*, known for facilitating protein–protein interactions, may provide valuable insights into the underlying mechanisms of resistance [46]. By concentrating on these novel targets, we can refine treatment strategies, ensuring the sustained efficacy of ACTs in combating malaria.

## 4. Materials and Methods

### 4.1. Blood Samples and Study Areas

**Figure 2:**
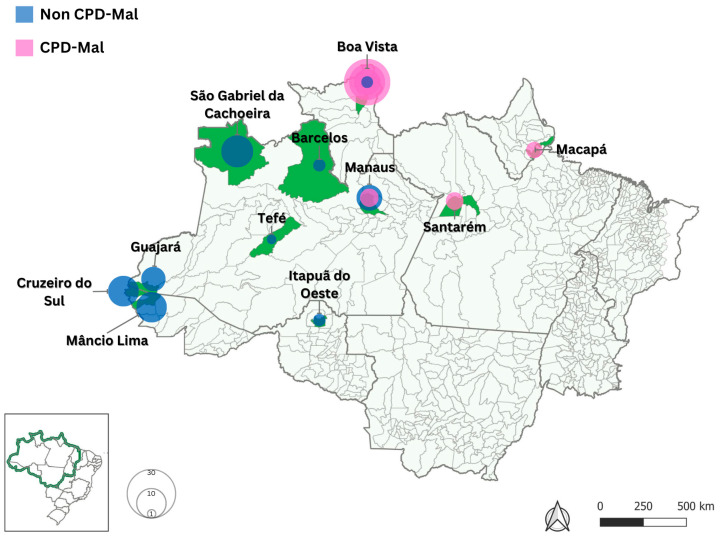
The spatial distribution of malaria cases is classified as the following: from the Reference Center for Research, Diagnosis, and Training of Malaria in non-endemic areas (CPD-Mal) or from the endemic Brazilian regions (non-CPD-Mal). The size of the circles represents the number of samples from each municipality. Green-shaded areas indicate the locations of infection.

Between 2013 and 2023, a total of 141 blood samples were collected from symptomatic patients diagnosed with *P. falciparum* infection (Figure 2). Of these, 30 samples were obtained from patients treated at the Acute Febrile Syndrome Outpatient Clinic of the Evandro Chagas National Institute of Infectious Diseases (INI-Fiocruz) in Rio de Janeiro, a member of the Reference Center for Research, Diagnosis, and Training of Malaria (CPD-Mal/Fiocruz) for the extra-Amazonian region (22°54′ S, 43°12′ W), led by the Malaria Research Laboratory. These CPD-Mal samples were distributed across six Brazilian states: Amazonas (twenty-three samples, including two from Barcelos in 2013 and 2016; six from Manaus in 2014, 2017, 2022, and 2023; fourteen from São Gabriel da Cachoeira between 2019 and 2023; and one from Tefé in 2014), Pará (three samples from Santarém in 2023), Rondônia (two samples from Itapuã do Oeste in 2014 and 2023), and Roraima (two samples from Boa Vista in 2022). All CPD-Mal patients were monitored for treatment outcomes, including parasite clearance rates, clinical progression, and molecular diagnosis [47,48,49,50], as per the guidelines established by the Brazilian Ministry of Health [4]. If the patients returned, they were evaluated on days 0, 1, 2, 3, 7, 14, 28, and 42; if symptoms appeared, they were assessed at any time during the follow-up period.

In addition to the CPD-Mal samples, 111 blood samples were collected from malaria-endemic regions across Brazil. In Amazonas, 23 samples were obtained, seven from Manaus (6 in 2013 and 1 in 2014) and 16 from Guajará in 2016. In Acre, 55 samples were collected, including 30 from Cruzeiro do Sul (20 in 2016 and 10 in 2018) and 25 from Mâncio Lima (12 in 2016 and 13 in 2018). In Roraima, 33 samples were obtained from Boa Vista, with 5 in 2016, 12 in 2021, and 16 in 2022. In Amapá, a single sample was collected from Macapá in 2019.

### 4.2. Ethical Aspects and Consent to Participate

The study protocol was approved by the Ethics and Research Committee for Research Involving Human Beings at Fiocruz (CAAE 88554718.0.3002.5248 and CAAE 46084015.1.0000.5248 for the Acre samples). Additionally, the Boa Vista samples were approved by the Research Ethics Committee of the Federal University of Roraima (CEP/UFRR) under CAAE 24122619.6.0000.5302.

All participants were thoroughly informed about the study protocols and procedures to ensure their understanding. Informed consent was obtained from each participant, either in writing or via thumbprint for those unable to sign, allowing the use of remnant blood samples. All study procedures strictly adhered to the federal regulations mandated by the Brazilian Ministry of Health.

### 4.3. Malaria Diagnosis and Nucleic Acid Extraction

The diagnosis of *P. falciparum* was initially performed on-site using light microscopy with a thick blood smear, regardless of the blood collection location. To confirm *P. falciparum* mono-infection, all samples underwent nucleic acid extraction and molecular diagnosis via polymerase chain reaction (PCR). For DNA extraction, 1 mL of the blood samples was processed and purified using the QIAamp^®^ DNA Mini kit, following the manufacturer’s instructions (Qiagen, Hilden, Germany). The extracted DNA was stored at −20 °C until PCR testing. Both conventional and real-time PCR assays were conducted using *Plasmodium*-specific primers [47]. Positive samples underwent further analysis with species-specific single or nested PCRs to detect *P. vivax* [48], *P. falciparum* [49], and/or *P. malariae* [50].

All samples were stored at the Malaria Research Laboratory (LPM) at Instituto Oswaldo Cruz (IOC), which serves as the headquarters for the Reference Center for Malaria Treatment and Diagnosis (CPD-Mal/Fiocruz). This study included only patients confirmed to have mono-infections of *P. falciparum.*

### 4.4. Gene Amplification and DNA Sequencing

Gene amplifications were conducted for *pfk13*, *pfmdr1*, and *pfcrt.* For *pfk13*, fragments of approximately 859 base pairs (bp) were amplified following the standard protocol [13]. Two fragments of the *pfcrt* gene were amplified: the first fragment, 145 bp, included exon 2 and covered positions 72–76, according to the protocol described by Zhou et al. (2016) [33]; the second fragment, 339 bp, included exon 10 and covered positions M343**L**, C350**R**, G353**V**, and I356**T**/**L**, as described by Foguim et al. (2020) [37]. For *pfmdr1*, amplification was carried out in two parts: a 501 bp fragment from the initial (start) region was amplified to analyze the SNPs N86**Y** and Y184**F.** Conversely, a 935 bp fragment from the terminal (end) region was used to assess the SNPs S1034**C**, N1042**D**, and D1246**Y**, following the previously described method [26]. The PCR products were analyzed using electrophoresis on a 2% agarose gel and visualized under a UV transilluminator (DigiDoc-It, UVP, Upland, CA, USA). Each PCR product was purified using the Wizard™ SV Gel and PCR Clean-Up System (Promega, Madison, WI, USA), following the manufacturer’s instructions. DNA sequencing was performed using the Big Dye™ Terminator Cycle Sequencing Ready Reaction version 3.1 (Applied Biosystems, Foster, CA, USA), with 3.2 μM of the forward and reverse PCR primers. DNA sequences were determined using the ABI Prism DNA Analyzer™ 3730 (Applied Biosystems, Foster, CA, USA) at the Fiocruz Genomic Platform PDTIS/Fiocruz RPT01A.

### 4.5. Data Analysis

Multiple nucleotide sequences were aligned using ClustalW within the free software BioEdit^®^ version 7.7.1 (North Carolina State University, Raleigh, NC, USA [51]). All mutations were assessed by comparing each sequence to the PF3D7_0709000 (*pfcrt*), PF3D7_0523000 (*pfmdr1*), and PF3D7_1343700 (*pfk13*) from PlasmoDB (http://www.plasmodb.org, accessed on 22 February 2025). The nucleotide sequences and their corresponding deduced amino acid sequences for each antimalarial drug resistance gene were further analyzed to detect polymorphisms and were compared to known resistance-associated mutations. Each haplotype’s allele frequencies and prevalence were estimated by the number of isolates carrying the specific haplotype and the total samples with successful sequencing.

All DNA sequences generated in this study have been deposited in the GenBank™ database (NIH genetic sequence database; www.ncbi.nlm.nih.gov/GenBank, accessed on 22 February 2025). The sequences for *pfk13* (PP584057–PP584104; PV172739–PV172845), *pfcrt* (exon 2: OQ672386–OQ672451 and PV172664–PV172738; exon 10: PV289785–PV289925), and *pfmdr1* (initial region: PV172846–PV172986; terminal region: PV605939–PV606079) are accessible under their respective accession numbers.

Genetic parameters such as haplotype diversity (Hd), nucleotide diversities, and the measures of neutrality (Tajima’s D, Fu and Li’s D*, and Fu and Li’s F*) were computed by DnaSP 6.12 [52]. In addition, intergenic and intragenic LD tests were performed by calculating the r2 values to determine the association between the SNPs of the three genes investigated using Haploview 4.1 software [53].

## 5. Conclusions

This study provided an updated overview of Brazil’s key indicators of *P. falciparum* resistance. The absence of *pfk13* resistance-associated mutations and the low prevalence of critical *pfmdr1* markers (N86Y and Y184F) supported the sustained efficacy of ACTs. The high frequency of *pfcrt* mutations and the dominance of the *pfmdr1* NYCDY haplotype suggested ongoing selective pressure and allele fixation driven by positive selection. The independent evolutionary paths of *pfcrt* and *pfmdr1* highlighted their distinct roles in drug resistance. The persistence of diverse haplotypes and intragenic linkage disequilibrium indicated that there has been continuous drug pressure. Additional studies, including in vitro susceptibility assays, could yield new insights into this topic. Ongoing genomic surveillance is crucial for tracking emerging resistance and informing adaptive, region-specific treatment strategies to maintain effective malaria control and delay the development of resistance.

## Figures and Tables

**Table 1:** Prevalence of *pfcrt* wild-type and mutant haplotypes in study areas.

Genotype ^1^	Samples N(%)	Brazilian States					
		Acre	Amazonas	Amapá	Roraima	Rondônia	Pará
		(55 s)	(46 s)	(1 s)	(34 s )	(2 s)	(3 s)
**S**VMN**T**MCGI **^2^**	83 (59)	44	27	1	7	2	2
**S**VMN**T**MCG**L ^3^**	56 (40)	11	17	0	27	0	1
CVMNKMCGI **^4^**	2 (1)	0	2	0	0	0	0

^1^ The bold and underlined characters represent a non-synonymous mutation. ^2^ S: codon 72; T: codon 76. ^3^ S: codon 72; T: codon 76; L: codon 356. ^4^ Reference Pf3D7 wild haplotype sequence; s = number of samples.

**Table 2:** Prevalence of *pfmdr1* wild-type and mutant haplotypes, according to Brazilian states.

Genotype ^1^	Number of Isolates (%)	Brazilian States					
		Acre	Amazonas	Amapá	Roraima	Rondônia	Pará
		(n = 55)	(n = 46)	(n = 1)	(n = 34)	(n = 2)	(n = 3)
NY**CD**D	16 (11)	12	4	0	0	0	0
NY**CDY ^2^**	98 (70)	35	27	1	31	1	3
NYSND **^3^**	19 (13)	8	9	0	2	0	0
NYSN**Y ^4^**	3 (2)	0	2	0	0	1	0
**Y**YSND ^5^	5 (4)	0	4	0	1	0	0

^1^ The bold and underlined characters represent a non-synonymous mutation. ^2^ C: codon 1034; D: codon 1042; Y: codon 1246. ^3^ Reference Pf3D7 wild haplotype sequence. ^4^ Y: codon 1246. ^5^ Y: codon 86.

**Table 3:** Distributions of *pfk13*, *pfcrt,* and *pfmdr1* combination alleles among 141 *P. falciparum* isolates from Brazilian endemic area.

Haplotypes ^1^			Combination Type	Samples N (%)
*pfk13*	*pfcrt*	*pfmdr1*		
Wild-type ^2^ +	**S**VMN**T**MCG**L**+	NY**CD**D	Quintuple	5 (4)
		NY**CDY**	Sextuple	44 (31)
		NYSND	Triple	5 (4)
		**Y**YSND	Quadruple	2 (1)
Wild-type ^2^ +	**S**VMN**T**MCGI+	NY**CD**D	Quadruple	10 (7)
		NY**CDY**	Quintuple	54 (38)
		NYSND	Double	13 (9)
		NYSN**Y**	Triple	3 (2)
		**Y**YSND	Triple	3 (2)
Wild-type ^2^ +	CVMNKMCGI ^2^ +	NY**CD**D	Double	1 (1)
		NYSND^2^	Wild	1 (1)

^1^ The bold and underlined characters represent a non-synonymous mutation. ^2^ Reference Pf3D7 wild haplotype sequence. “+” indicates the co-occurrence of specific allelic variants in *pfk13*, *pfcrt*, and *pfmdr1* within the same *P. falciparum* isolate, representing a multilocus haplotype.

**Table 4:** Genetic diversity parameters of *pfcrt*, *pfmdr1,* and *pfk13* genes.

Parameters		*pfcrt*		*pfmdr1*		*pfk13*
		Exon 2	Exon 10	Initial Region	Terminal Region	Helix Domain
SNPs (n)		2	2	1	3	0
Haplotypes (n)		3	2	2	4	1
Nucleotide diversity	π	0.00182	0.00165	0.00012	0.00112	0
	θ	0.00249	0.00063	0.00033	0.00058	0
Haplotype diversity		0.146	0.471	0.068	0.483	0
Variance Hd		0.00152	0.00045	0.00083	0.00184	0
SD Hd		0.039	0.021	0.029	0.043	0
**Neutrality Tests**						
Tajima’s D		−0.39181	1.72185	−0.66673	1.58461	0
Fu and Li D *		0.65945	0.4719	0.4719	0.79831	0
Fu and Li F *		0.38727	0.9992	0.1451	1.2374	0

π: Pi; θ: Theta; Variance Hd: variance of the haplotype diversity; SD Hd: standard deviation of the haplotype diversity. For the neutrality tests, the *p*-value was >0.10 for all genes. * Fu and Li’s D and F* statistics were calculated using an outgroup sequence.

## Data Availability

Data supporting the conclusions of this article are included within the article. The datasets used and/or analyzed during the present study are available from the corresponding author on reasonable request.
